# The changing epidemiology of diphtheria in the United Kingdom, 2009 to 2017

**DOI:** 10.2807/1560-7917.ES.2020.25.11.1900462

**Published:** 2020-03-19

**Authors:** Charlotte M Gower, Antonia Scobie, Norman K Fry, David J Litt, J Claire Cameron, Meera A Chand, Colin S Brown, Sarah Collins, Joanne M White, Mary E Ramsay, Gayatri Amirthalingam

**Affiliations:** 1 Immunisation and Countermeasures Division, National Infection Service, Public Health England, London, United Kingdom; 2 These authors contributed equally to this work; 3 Vaccine Preventable Bacteria Section, National Infection Service Laboratories, Public Health England, London, United Kingdom; 4 NHS National Services Scotland, Health Protection Scotland, Meridan Court, Glasgow, United Kingdom; 5 Department of Infection, Royal Free London NHS Foundation Trust, London, United Kingdom

**Keywords:** Corynebacterium, ulcerans, diphtheriae, cutaneous, respiratory, vaccination

## Abstract

**Background:**

Diphtheria is a potentially fatal disease caused by toxigenic strains of *Corynebacterium diphtheriae, C. ulcerans* or *C. pseudotuberculosis.*

**Aim:**

Our objective was to review the epidemiology of diphtheria in the United Kingdom (UK) and the impact of recent changes in public health management and surveillance.

**Methods:**

Putative human toxigenic diphtheria isolates in the UK are sent for species confirmation and toxigenicity testing to the National Reference Laboratory. Clinical, epidemiological and microbiological information for toxigenic cases between 2009 and 2017 are described in this population-based prospective surveillance study.

**Results:**

There were 33 toxigenic cases of diphtheria aged 4 to 82 years. Causative species were *C. diphtheriae* (n = 18) and *C. ulcerans* (n = 15). Most *C. diphtheriae* cases were cutaneous (14/18) while more than half of *C. ulcerans* cases had respiratory presentations (8/15). Two thirds (23/33) of cases were inadequately immunised. Two cases with *C. ulcerans* infections died, both inadequately immunised. The major risk factor for *C. diphtheriae* aquisition was travel to an endemic area and for *C. ulcerans,* contact with a companion animal. Most confirmed *C. diphtheriae* or *C. ulcerans* isolates (441/507; 87%) submitted for toxigenicity testing were non-toxigenic*,* however, toxin positivity rates were higher (15/23) for *C. ulcerans* than *C. diphtheriae* (18/469). Ten non-toxigenic toxin gene-bearing (NTTB) *C. diphtheriae* were also detected.

**Conclusion:**

Diphtheria is a rare disease in the UK. In the last decade, milder cutaneous *C. diphtheriae* cases have become more frequent. Incomplete vaccination status was strongly associated with the risk of hospitalisation and death.

## Introduction

Diphtheria was once one of the most feared childhood diseases in the United Kingdom (UK), with ca 60,000 cases per year, but dramatically reduced following introduction of mass immunisation in 1942 [1]. Diphtheria vaccine is made from inactivated diphtheria toxin and protects individuals from the effects of toxin-producing corynebacteria. In the UK, diphtheria toxoid is included in the immunisation schedule at 2, 3 and 4 months of age followed by two boosters (at approximately 3 and 14 years of age), with further boosters recommended for travel and as part of the maternal pertussis immunisation programme following inclusion in the pertussis booster vaccine [1]. In addition, CRM_197_-containing vaccines (a non-toxigenic mutant of diphtheria toxin used as a carrier protein in some conjugate vaccines such as pneumococcal conjugate vaccine), provide additional boosting of diphtheria antibodies in the population [2]. 

Diphtheria vaccine coverage in the UK remains high; coverage of the primary course evaluated at 1 and 2 years of age has been between 91% and 95% since the early 1990s. Assessment of preschool booster coverage started in 1999/2000; coverage remained between 78% and 82% during the following decade, before increasing to 86% in 2009/2010 and remaining between 86% and 89% since [3]. Coverage assessment of the adolescent booster began in 2016, and is ca 85% [4]. The last UK seroprevalence study in 2009 found that 75% of the population had antibody levels ≥ 0.01 IU/mL, correlating with basic diphtheria protection [2]. The highest number of susceptible individuals were observed in under 1-year-olds (37%), 35–44-year-olds (27%), 45–69-year-olds (41%) and those older than 70 years (33%), perhaps reflecting a combination of waning immunity and fewer doses offered in earlier schedules [2].

Three *Corynebacterium* species can potentially produce diphtheria exotoxin; *C. diphtheriae* (associated with epidemic person-to-person spread via respiratory droplets and close contact), *C. ulcerans* (traditionally associated with farm animal contact and dairy products) and *C. pseudotuberculosis* (which rarely infects humans and is typically associated with farm animals, particularly ruminants) [5,6]. Classical respiratory diphtheria is characterised by the formation of an adherent grey-white pseudomembrane in the throat [1,7]. Milder respiratory disease may manifest as a sore throat, and is most commonly seen in fully or partially vaccinated individuals. Diphtheria may also present with cutaneous lesions, characterised by rolled-edge ulcers usually on exposed limbs, particularly the legs, and is more common in tropical regions. The mode of transmission of cutaneous diphtheria is through direct contact with lesions or infected secretions with some evidence suggesting it may be more transmissible than respiratory diphtheria [7]. Patients may exhibit both cutaneous and respiratory disease at the same time, whether caused by *C. diphtheriae or C. ulcerans* [8]. *C. ulcerans* has a broad host range and has been isolated from clinically affected and healthy wild, farm, zoo, research and domesticated animals, as reviewed by Tiwari et al. [9]. Severe clinical cases of respiratory diphtheria require rapid administration of diphtheria antitoxin (a concentrated immunoglobulin preparation prepared from horse serum, that neutralises circulating toxin), as well as antibiotics to clear the bacterial infection [8]. However, economic factors as well as issues concerning regulations have led to poor availability of diphtheria antitoxin (DAT) in many countries [10,11], although it has been continuously available in the UK in limited supplies.

Revised guidelines for public health management and control of diphtheria in England published in 2015 included the availability of a rapid national PCR service for confirmation of identification of the toxigenic *Corynebacterium* species and detection of the toxin gene (*tox*) in addition to the phenotypic Elek test, to enable faster public health action [7]. The availability of both PCR and Elek testing has identified a number of *C. diphtheriae* isolates carrying the *tox* gene (PCR-positive) but not expressing the toxin (Elek-negative), termed non-toxigenic toxin gene-bearing strains (NTTB) [12]. The pathogenesis and clinical significance of isolation of this organism are not well understood; in particular they are not known to cause diphtheria (which is due to action of the toxin) and patients are therefore not treated with antitoxin. However, owing to their potential (currently unquantifiable) risk of becoming toxigenic through a genetic event, it is currently recommended in the UK that they are managed in the same way as as fully toxigenic (i.e. Elek-positive, toxin-expressing) diphtheria cases and eliminated using antibiotics from patients and contacts [7]. 

The last review of UK diphtheria cases from 1986 to 2008 highlighted the changing epidemiology with two-thirds of toxigenic infections being indigenously acquired *C. ulcerans* rather than imported *C. diphtheriae*. Similar trends of increasing *C. ulcerans* cases were reported in other western European countries [5]. We present an updated epidemiological analysis of diphtheria in the UK based on surveillance and laboratory testing of all *C. diphtheriae* and *C. ulcerans* isolates from 2009 until 2017 and following the availability of a rapid national PCR service to assess the impact of changes in the surveillance system and inform public health risk assessment and practice.

## Methods

It is a legal requirement in the UK that all human isolates of *C. diphtheriae*, *C. ulcerans* and *C. pseudotuberculosis* identified by microbiological laboratories are reported to public health authorities and isolates should be sent for toxigenicity testing to the National Reference Laboratory (NRL) for diphtheria, part of National Infection Service at Public Health England (PHE), Colindale [7]. Since April 2014, all submitted isolates have been routinely tested by real-time PCR [12], to detect *C. diphtheriae* and *C. ulcerans/pseudotuberculosis*, and the presence or absence of the *tox* gene. Differentiation between *C. ulcerans and C. pseudotuberculosis* is made using phenotypic testing and confirmation of expression of diphtheria toxin in PCR *tox*-positive isolates is sought using the modified Elek test [12]. Until April 2014, characterisation of pathogenic corynebacteria was undertaken using traditional phenotypic methods only, and detection of toxin by Elek testing [13].

We conducted a prospective population-based surveillance study of diphtheria in the UK between 2009 and 2017. Information on all UK isolates received by the NRL for species confirmation and toxigenicity testing between January 2009 and December 2017 was extracted from PHE laboratory records. Completeness of submissions was investigated using the second-generation surveillance system (SGSS) – an electronic laboratory reporting system for clinically significant infections for hospital laboratories to PHE.

### Diphtheria cases in the United Kingdom

All cases of toxigenic (demonstrated by detection of toxin gene by PCR and Elek positivity) *C. diphtheriae* and *C. ulcerans* and NTTB (*tox*-positive by PCR, Elek-negative) *C. diphtheriae* infections are reported to local Health Protection teams who conduct public health management of cases, with support from the national centres: Health Protection Scotland (HPS), Public Health Wales (PHW), Public Health Northern Ireland (PHNI) and PHE. Clinical, epidemiological and microbiological information were collected on all UK cases between January 2009 and December 2017.

Data on vaccination status was collected from primary care records and occasionally based on patient recall. Patients were defined as vaccinated if they had received the required five doses of a diphtheria toxoid, or the appropriate number of doses for their age, according to the UK immunisation schedule [1]. Cases where there was no record of vaccination were considered as inadequately vaccinated. It may be, particularly for older individuals who had moved practices during their life, that they had received vaccinations as children but these doses were not recorded in current records, but in general, primary care records are very detailed, and it is more likely that individuals without recorded vaccinations were under-immunised. Numbers of cases before and after the introduction of the 2015 guidelines and PCR testing were compared. The 2015 guidelines were initially introduced as interim guidelines in April 2014 (at the time of the introduction of the PCR testing) and thus the comparison is made before and after April 2014.

### Ethical statement

The data presented in this review were collected as a part of routine national surveillance for vaccine preventable diseases by PHE and HPS. In accordance with Regulation 3 of the Health Service (Control of Patient Information) Regulations 2002, which allows confidential information to be processed for national surveillance of communicable diseases, individual patient consent was not required.

## Results

### Testing of putative *Corynebacterium* *diphtheriae* and *Corynebacterium ulcerans* isolates 2009–2017

**Figure 1:**
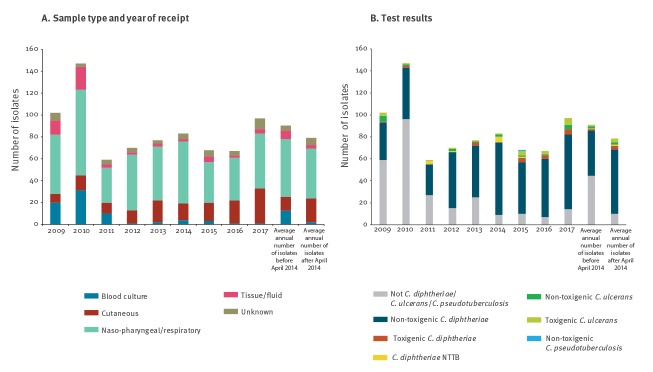
Testing of all putative *Corynebacterium diphtheriae* and *Corynebacterium ulcerans* received by the National Reference Laboratory for confirmation and toxigenicity testing, United Kingdom, 2009–2017 (n = 770)

The NRL tested 770 putative *C. diphtheriae* or *C. ulcerans/C. pseudotuberculosi*s isolates in the UK between 2009 and 2017, representing more than 98% of cases recorded on SGSS. Among those, 98% (758/770) were from human patients and 2% (12/770) from animals (usually animal contacts of a human toxigenic *C. ulcerans* case). Of the 770 samples, 700 were from England (including nine animals), 47 from Scotland (including three animals), 19 from Wales and four from Northern Ireland. There was no notable change in the average annual number of isolates tested following PCR introduction (Figure 1). More than half of the samples (58%) were oral or respiratory-tract samples. Since 2014, there has been a reduction in the number of referred blood cultures, with an increase in referred cutaneous specimens (e.g. wound swabs) from 13% before April 2014 to 28% after April 2014 (Figure 1A).

NTTB: non-toxigenic toxin gene-bearing.

An average annual number of isolates is presented before and after April 2014 when PCR testing was introduced.

Of all isolates tested, 61% (469/770) were *C. diphtheriae*, 5% (38/770) were *C. ulcerans*, 0.1% (1/770) were *C. pseudotuberculosis* and 34% (262/770) were not *C. diphtheriae*, *C. ulcerans or C. pseudotuberculosis* (Figure 1B). The number of isolates that were not *C. diphtheriae, C. ulcerans or C. pseudotuberculosis* reduced substantially over time. Although most isolates were non-toxigenic, the proportion of toxigenic isolates (PCR- and Elek-positive) was substantially higher for *C. ulcerans* (55%, 21/38) than *C*. *diphtheriae* (4%, 18/469). The higher percentage of toxigenic *C. ulcerans* isolates cannot be explained by the presence of linked results for companion animal, as a similar pattern was observed when the dataset was restricted to human cases (58%; 15/26). The percentage of isolates that were toxigenic varied by year, with an apparent increase in the average percentage of both *C. diphtheriae* and *C. ulcerans* toxigenic strains following the introduction of PCR testing (before April 2014: *C. diphtheriae* 2.56% *C. ulcerans* 43.8%; after April 2014: *C. diphtheriae* 5.10%; *C. ulcerans* 63.6%).

### Laboratory-confirmed diphtheria cases and non-toxigenic toxin-bearing *Corynebacterium* isolates in the United Kingdom 2009–2017

**Figure 2:**
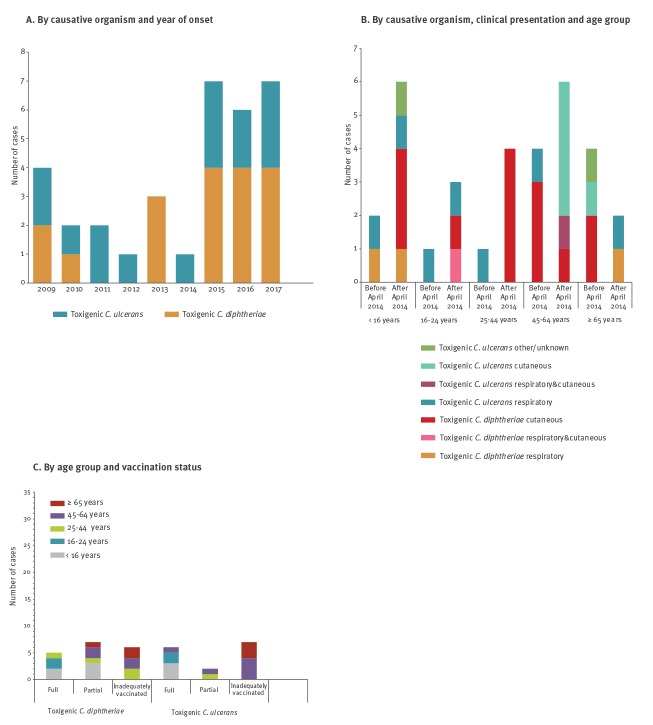
Toxigenic diphtheria cases, United Kingdom, 2009–2017 (n = 33)

During the study period, 33 cases of human toxigenic diphtheria were identified in the UK (three in Scotland; 30 in England; 0 in Northern Ireland; 0 in Wales) with one to seven cases per year (median: three cases): 18 *C. diphtheriae* and 15 *C. ulcerans* (Figure 2A). The age range of cases was 4–82 years and 19 of 33 were female. The incidence of toxigenic diphtheria was higher after introduction of PCR testing in 2014 with an overall increase from 0.0045 to 0.0089 cases per 100,000 population per year. The predominant cause of toxigenic cases was *C. diphtheriae,* with a greater increase after 2014 for *C. diphtheriae* compared with *C. ulcerans* (average annual incidence increased from 0.0022 per 100,000 population (before April 2014) to 0.0049 per 100,000 population after April 2014 for toxin-producing *C. diphtheriae* and 0.0022 to 0.0037 cases per 100,000 population per year for *C. ulcerans* cases).

Please note the time period before April 2014 and after April 2014 are not equivalent.

In addition, 10 cases with NTTB *C. diphtheriae* strains were identified including four cases from before April 2014 following retrospective testing during the PCR validation phase [14]. A cluster of five NTTB was observed in 2014 by active screening following identification in two patients attending the same dermatology clinic, and all cases had underlying chronic skin lesions. A further unrelated case was documented in 2015.

There was no overall difference in age or sex of *C. diphtheriae* or *C. ulcerans* cases; however, NTTB *C. diphtheriae* cases were more frequent in younger age groups (Table 1) and in male rather than female patients (*C. diphtheriae*: 8/18 male, *C. ulcerans*: 6/15 male, NTTB: 7/10 male). Mean age was 35.7 years (± 95% confidence interval (CI): 11.8) for toxigenic *C. diphtheriae* cases; 43.1 years (± 95% CI: 12.5) for toxigenic *C. ulcerans* cases and 27.9 years (± 95% CI: 3.3) for NTTB cases.

**Table 1:** Diphtheria cases by species, clinical presentation and age group, United Kingdom, 2009–2017 (n = 43)

	< 16	16–24	25–44	45–64	≥ 65	Total
**Toxigenic *Corynebacterium diphtheriae***	**5**	**2**	**4**	**4**	**3**	**18**
Cutaneous	3	1	4	4	2	14
Respiratory (no membrane)	1	0	0	0	1	2
Respiratory (no membrane)/cutaneous	0	1	0	0	0	1
Respiratory (with membrane)	1	0	0	0	0	1
**Toxigenic *Corynebacterium ulcerans***	**3**	**2**	**1**	**6**	**3**	**15**
Cutaneous	0	0	0	4	1	5
Other/unspecified	1	0	0	0	1	2
Respiratory (no membrane)	2	2	1	1	0	6
Respiratory (with membrane)	0	0	0	0	1^a^	1
Respiratory (with membrane)/cutaneous	0	0	0	1^a^	0	1
**Total toxigenic cases**	**8**	**4**	**5**	**10**	**6**	**33**
***Corynebacterium diphtheriae* NTTB**	0	**3**	**7**	0	0	**10**
Asymptomatic	0	1	2	0	0	3
Cutaneous	0	1	3	0	0	4
Respiratory (no membrane)	0	1	2	0	0	3

^a^ Fatality.

### Clinical and epidemiological characteristics

#### Diphtheria cases

Of the 33 toxigenic cases, 19 were cutaneous, 10 were respiratory, two had both respiratory and cutaneous symptoms and two had other presentations (Table 1). Most toxigenic *C. diphtheriae* cases were cutaneous (14/18), although there were two mild respiratory cases and one case of classical respiratory diphtheria. A further case had both cutaneous and mild respiratory disease. Conversely, more than 50% (8/15) of *C. ulcerans* cases had respiratory presentations, with two having classical symptoms including a membrane.

The percentage of cutaneous cases increased over time, being nearly three times higher after 2014 compared with the preceding 3 years (Figure 2B). This was seen for both *C. diphtheriae* and *C. ulcerans.* There were no differences in clinical presentation by age group (Table 1) or sex (10 of 19 cutaneous cases and 7 of 13 respiratory cases were male). Similarly, there was no evidence of an interaction between age (Table 1), sex, clinical presentation and *Corynebacterium* species (data not shown) in the toxigenic cases; however, after 2014, toxigenic *C. diphtheriae* was increasingly detected in younger age groups and *C. ulcerans* in older age groups (Figure 2B).

Eleven of 33 cases were hospitalised, 15 of 33 were not admitted and for seven of 33, this information was not recorded. For those with known hospitalisation status, *C. diphtheriae* cases (6/15) and *C. ulcerans* cases (5/11) had similar hospitalisation rates. Although overall cases numbers were small, it appears that cases were more likely to be hospitalised before April 2014 (*C. diphtheriae*: 2/3; *C. ulcerans*: 2/3) than after April 2014 (*C. diphtheriae*: 4/12; *C. ulcerans*: 3/8). Between 2009 and 2017, there were two deaths, both with *C. ulcerans* infection in individuals with inadequate vaccination status in the 50–64-year age group, giving an overall diphtheria case fatality rate of 6% (13% for *C. ulcerans*). The case fatality for diphtheria cases with respiratory presentation was 15% (2/13). One fatal case presented with a membrane and cutaneous disease, the other without a membrane, and neither received DAT. There were two *C. diphtheriae* cases with systemic complications - a partially vaccinated child with classical respiratory diphtheria and multisystem complications who received DAT, and an additional case of unknown vaccination status with cutaneous disease, who did not receive DAT but also had invasive Lancefield group A streptococcal infection. Only one appropriately vaccinated case was hospitalised (1/11) compared with 10 of 22 inadequately vaccinated individuals.

Overall, 22 of 33 cases were inadequately vaccinated for their age (including those with unknown or unclear vaccination status), 13 of 18 *C. diphtheriae* cases and nine of 15 *C. ulcerans* cases (Table 2). Of the 11 appropriately vaccinated cases, five presented with cutaneous disease, four had mild respiratory disease, one had a combination of respiratory and cutaneous symptoms and one had an ‘other’ presentation (Table 2). Vaccinated cases were more commonly from younger age groups (Figure 2C). There was an increase in fully vaccinated cases following the change in the surveillance programme, with only two of 12 cases being fully vaccinated before April 2014 compared with nine of 21 cases after.

**Table 2:** Diphtheria cases by species, clinical presentation and vaccination status, United Kingdom, 2009–2017 (n = 43)

	Fully vaccinated for age	Partially vaccinated	Not vaccinated	Unknown or unclear vaccination status	Total
**Toxigenic *Corynebacterium diphtheriae***	**5**	**7**	**1**	**5**	**18**
Cutaneous	4	5	0	5	14
Respiratory (no membrane)	0	1	1	0	2
Respiratory (no membrane)/cutaneous	1	0	0	0	1
Respiratory (with membrane)	0	1	0	0	1
**Toxigenic *Corynebacteriumulcerans***	**6**	**2**	**1**	**6**	**15**
Cutaneous	1	1	0	3	5
Other/unspecified	1	0	0	1	2
Respiratory (no membrane)	4	1	0	1^a^	6
Respiratory (with membrane)	0	0	1^a^	0	1
Respiratory (with membrane)/cutaneous	0	0	0	1	1
**Total toxigenic cases**	**11**	**9**	**2**	**11**	**33**
***Corynebacterium diphtheriae* NTTB**	**4**	0	0	**6**	**10**
Asymptomatic	1	0	0	0	1
Cutaneous	1	0	0	3	4
Respiratory (no membrane)	2	0	0	1	3
Unknown	0	0	0	2	2

NTTB: non-toxigenic toxin gene-bearing.

^a^ Fatality.

All 33 cases were treated with macrolide antibiotics. A post-infection booster dose of diphtheria toxoid-containing vaccine is recommended for all diphtheria cases once they are clinically stable [7] since infection does not always induce adequate levels of immunity and 12 of 33 had a documented date of post-infection immunisation in their public health records. All cases were followed up for close contacts with an average of 21.4 total contacts per case (range: 0–154). The average number of household contacts was 2.5 per case (range: 0–6) and of healthcare worker (HCW) contacts 8.7 per case (range: 0–122). At least five cases had more than 30 HCW contacts. All contacts were swabbed and chemoprophylaxis and vaccination of contacts was documented for 28 of 33 cases. One contact of a cutaneous case was positive for toxigenic *C. diphtheriae*.

The major risk factor for infection with toxigenic *C. diphtheriae* was travel to an endemic country in Africa, Asia or Oceania within the previous 3 months, with 14 of 18 cases characterised as imported (Table 3); all imported cases presented with cutaneous disease. Three of the 18 cases, including the classical respiratory case, had no known history of travel or contact with someone who had travelled to an endemic region and their method of acquisition remains unknown. One mild respiratory case was a close contact of an imported cutaneous *C. diphtheriae* case, but had not herself travelled.

**Table 3:** Identified risk factors of diphtheria cases and non-toxigenic toxin-bearing *Corynebacterium* *diphtheriae* isolates, United Kingdom, 2009–2017 (n = 43)

Risk group	NTTB	Toxigenic *Corynebacterium diphtheriae*	Toxigenic *Corynebacterium* *ulcerans*
Animal contact^a^	0	0	15
Contact of case	0	1	0
Underlying skin condition	5	0	0
Travel^b^	0	14	0
None known	5	3	0

NTTB: non-toxigenic toxin gene-bearing.

^a^ Contact reported with dogs (12 cases), cats (7 cases), rabbits (3 cases), horses (2 cases), sheep (1 case) and guinea pigs (1 case).

^b^The suspected countries of importation were Samoa, India, Pakistan, Sri Lanka, Thailand, The Philippines (3 cases), Congo, Ghana (2 cases), Sierra Leone (2 cases), and Somalia

The major risk factor for *C. ulcerans* infection was exposure to domestic animals, with all cases reporting exposure to either dogs (12/15) or cats (7/15) (Table 3). Exposure to rabbits, horses and guinea pigs was also reported. Only one case had contact with farm animals (sheep), with no reported contact with cattle although one case did have a history of drinking unpasteurised milk. One case reported recent travel to Slovenia, but no other *C. ulcerans* cases had a recent history of travel. Swabs were taken from companion animals for nine cases, most commonly from dogs and cats; in three cases, at least one dog screened positive for toxigenic *C. ulcerans. C. ulcerans* was not detected in any of the other 14 animals that underwent screening.

#### Non-toxigenic toxin gene-bearing cases

The NTTB cases were both cutaneous (5/10) and respiratory (5/10) in presentation, although the cutaneous cases were all from one cluster and all had a chronic underlying dermatological condition, thus it was not clear if their symptoms were due to the *C. diphtheriae* NTTB infection. Two of the cases from the cluster were fully vaccinated, and three had unknown vaccination status. One case was hospitalised. Limited epidemiological and clinical information was available for the four cases that were identified retrospectively, but these four, and the remaining case identified outside of the cluster, all had a mild respiratory presentation and were young men without a history of travel. NTTB cases were managed as toxigenic cases. All six cases for whom this information was available received macrolide antibiotics and four of six had a documented date of post-infection vaccination. Contact tracing was conducted for all six cases identified as NTTB at the time, with an average of six contacts per case (range: 1–9). All contacts were swabbed, vaccinated and treated with a macrolide for 7 days.

## Discussion

The overall incidence of diphtheriae in the UK has remained low with a median of three cases per year, reflecting the success of the vaccination programme. A major change was a large increase in the proportion of cutaneous diphtheria cases, particularly caused by *C. diphtheriae*, which resulted in a slight predominance of this species as a causative agent in contrast with a previous review of UK cases between 1986 and 2008 which had reported greater numbers of *C. ulcerans* cases, as had also been reported in other western European countries during the same period [3,8]. During our study period, *C. ulcerans* continued to be an important pathogen and was responsible for the only two fatalities. Case fatality was low, at 6% overall and 9% in respiratory cases. At least half of all cases, and more than 70% of *C. diphtheriae* cases, were cutaneous in presentation, in contrast to less than 20% of all toxigenic cases, or 24% of *C. diphtheriae* cases, between 1986 and 2008 [8]. There was a significant increase in the proportion of referred isolates from wounds before and after 2014, suggesting that the increased detection of cutaneous cases could be due to changes in testing policies at frontline laboratories; this may be a consequence of the new guidelines but may also reflect unrelated changes such as the increased use the use of matrix-associated time of flight mass spectroscopy (MALDI-TOF) in place of culture for all routine wound swabs. Our results are similar to other recent reports in European countries of a rare but increasingly detected disease, such as in Belgium, which was disease-free from 1990 until 2010 but has reported 15 cases of toxigenic diphtheria since 2015, two-thirds of which were cutaneous in presentation [15]. In Belgium, no *C. diphtheriae* cases were reported between 2010 and 2015, but since 2015, although the majority of cases were still *C. ulcerans*, there have also been four *C. diphtheriae* cases, including a fatal case in an unvaccinated 3-year-old child. 

The major risk factor for diphtheria was inadequate immunisation, with two thirds of cases (22/33) without evidence of appropriate immunisation for age. Toxigenic cases, both cutaneous and mild respiratory presentations, also occurred in fully vaccinated individuals, but risk of hospitalisation and death were strongly related to inadequate immunisation. Although there was a high proportion (approximately a third) of cases occurring in fully vaccinated individuals, this was expected given the historically high vaccination coverage rate which means that most of the UK population is vaccinated. Travel to an endemic area was the primary risk factor for acquisition of toxigenic *C. diphtheriae*, and all toxigenic cutaneous diphtheria cases had travelled to countries in Africa, Asia or Oceania. Notably, three respiratory *C. diphtheriae* cases, including a case of classical respiratory diphtheria, had no known contacts or travel history and the method of acquisition remains unknown. The remaining respiratory case had recent contact with a confirmed cutaneous case that had recently travelled, representing the first domestic transmission of *C. diphtheriae* in the UK for over three decades [16]. Exposure to cats or dogs continued to be the risk factor for infection with *C. ulcerans*, as reported for the first time in the last review of UK diphtheria cases [8]. Of the companion animals that underwent screening in the UK during this period, the organism was only isolated from domestic dogs [17]. A recent survey in the Osaka district in Japan reported a prevalence of *C. ulcerans of* 7.5% among 583 asymptomatic dogs [18], however, similar information on prevalence of carriage and impact of *C. ulcerans* on animal health in the UK is not available. There was no evidence of an link with farm animals or unpasteurised dairy products, as seen historically.

Non-toxigenic *C. diphtheriae* usually lack the entire *tox* operon, however, a small proportion of non-toxigenic strains carry incomplete *tox* variants but do not express the diphtheria toxin protein, and are designated NTTB [14]. Routine identification of infections with NTTB are a more recently reported phenomenon in the UK since the introduction of PCR testing; however the pathogenesis and clinical significance of isolation of this organism is less well understood, and carriage is not likely to be influenced by vaccination, which targets the diphtheria toxin.

Analysis of the total isolates submitted for species identification and toxigenicity highlighted a significant difference in toxigenicity rates between *C. diphtheriae* and *C. ulcerans*, with ca 5% of *C. diphtheriae* and 50–60% of *C. ulcerans* isolates being toxin-producing, although we are unsure of the underlying mechanisms involved. One hypothesis is that the prevalence of the toxin gene is very high in the *C. ulcerans* population in UK animal hosts, however no systematic survey has been undertaken to confirm this. Since submissions to the NRL are based on isolates from symptomatic cases, they are not useful for estimation of the overall carriage rate of non-toxigenic corynebacteria in the UK, but a minimum incidence rate of carriage in symptomatic cases of 0.73 cases per 100,000 population per year was estimated, which is in line with estimates from other European countries [18].

### Strengths and limitations

The strength of this study is the high quality of the national surveillance programmes with active case management of all toxigenic cases and a statutory requirement for all laboratories to report cases of *C. diphtheriae* and *C. ulcerans* to public health authorities. The NRL at PHE is the only laboratory providing toxigenicity testing in the UK, and electronic reporting systems confirmed the completeness of submissions from frontline laboratories. A limitation of the study is the small sample size of cases which cautions against interpreting significant epidemiological trends.

### Implications for policy and surveillance

The data presented here regarding UK diphtheria cases in the last decade confirm the relative and increasing absolute and relative rate of cutaneous diphtheria, particularly in young adults with a history of travel and the potential for transmission to vulnerable contacts. Public health professionals should be aware that both cutaneous and respiratory cases of either *C. diphtheriae* or *C. ulcerans* have occurred in fully vaccinated individuals, although vaccination is still highly protective against severe disease. Inadequately vaccinated individuals are most at risk of severe or fatal disease and need prompt management, particularly assessment for diphtheria antitoxin. Despite limited global supplies of diphtheria antitoxin worldwide, the UK has not experienced shortages which have impacted on the clinical management of cases during the period of this study; however, DAT has not always been administered even in severe cases which may reflect a lack of of clinicians’ awareness of diphtheria given the rarity of the disease. To prevent sequelae or fatal outcome, prompt administration of diphtheria antitoxin (DAT) (within 24–48 h) is critical and it is currently recommended when there is clinical suspicion of classical respiratory diphtheria [7]. *Corynebacterium ulcerans* continues to present a serious threat, especially to older inadequately vaccinated individuals, and is a zoonotic risk from companion animals. The documented transmission of a toxigenic strain from a fully vaccinated individual to an unvaccinated contact reinforces the importance of identifying and screening all close contacts and maintaining high vaccine coverage to protect vulnerable individuals. However, delays in diagnosis have led in some cases to the need to exclude significant numbers of healthcare workers from clinical duties, with potential impact on the delivery of services.

Effective management of a *C. ulcerans* case requires coordination between human and animal health agencies. Hogg et al. raise several ethical and practical issues, including the different licensing of antibiotic types for human and veterinary use and the lack of legal compulsion for owners to treat companion animals found to be harbouring a toxigenic *C. ulcerans* strain, particularly when the animals themselves are non-symptomatic [15]. Public health management of human contacts of animal index cases may also be required. Analysis of testing data has demonstrated a much higher rate of toxigenicity of *C. ulcerans* among the submitted isolates and this could also have implications for early risk assessment and actions while awaiting toxigenicity testing results, although it is generally reported that *C. ulcerans* is less transmissible than *C. diphtheriae* [7].

## Conclusion

Although diphtheria is rare in the UK, it can cause severe and potentially fatal disease in inadequately immunised individuals, and clinicians should be aware of the need for early assessment for antitoxin in suspected cases. There is a risk of *C. diphtheriae* acquisition from travel to endemic regions, and *C. ulcerans* continues to emerge as an indigenous zoonotic pathogen in the UK. The low overall incidence of diphtheria in the UK population, and the shift towards identifying milder cutaneous disease demonstrate the continued success and importance of the diphtheria vaccination and surveillance programme; sustaining high vaccination coverage remains a key priority.
